# Mating Disruption of the Olive Moth *Prays oleae* (Bernard) in Olive Groves Using Aerosol Dispensers

**DOI:** 10.3390/insects12121113

**Published:** 2021-12-13

**Authors:** Antonio Ortiz, Andrés Porras, Jordi Marti, Antonio Tudela, Álvaro Rodríguez-González, Paolo Sambado

**Affiliations:** 1 Department of Organic and Inorganic Chemistry, EPS Linares, University of Jaén, Avda, Universidad, 23700 Linares, Spain; 2 Biogard, CBC Iberia, Avinguda Diagonal, 605, 08028 Barcelona, Spain; andres.porras@cbciberia.es (A.P.); jordi.marti@cbciberia.es (J.M.); paolo.sambado@cbciberia.es (P.S.); 3 Bioensayos y Experiencias Agrícolas S.L, 23001 Jaén, Spain; bioensayos.sl@gmail.com; 4 Instituto de Medio Ambiente, Recursos Naturales y Biodiversidad, Escuela de Ingeniería Agraria y Forestal (EIAF), Universidad de León, Avenida de Portugal 41, 24071 León, Spain; alrog@unileon.es

**Keywords:** insect sex pheromones, olive pest, integrated pest management, aerosol devices

## Abstract

**Simple Summary:**

According to official data, the world production of olive oil in the 2020–2021 period was about 3,197,000 tons; in the EU alone, the number of olive trees is over 737 million. The olive moth *Prays oleae* is one of the most damaging and devastating olive tree pests in the Mediterranean basin. Damage caused by this moth can reduce production by 50–60%, causing large losses in olive oil production. The use of an insect pheromone mating disruption strategy is a sustainable and environmentally friendly tool for Integrated Pest Management, reducing the use of chemical pesticides to control olive pests. In the present study, a mating disruption system based on aerosol dispensers was developed to control *P. oleae* moth populations. Overall, our results demonstrated at all experimental sites over 2 years that mating disruption using aerosols in the management of *P. oleae* registered a high suppression of male captures, as well as significantly reducing affected inflorescence and fruit infestation when compared to the untreated control.

**Abstract:**

The olive moth (OM), *Prays oleae* (Bern.) (Lepidoptera: Yponomeutidae), is a major olive grove pest worldwide; however, until now, very few studies have investigated the effectiveness of mating disruption (MD) techniques against this pest. Experiments were carried out for two successive years (2019 and 2020) in three different olive groves in Andalucía (Southern Spain) to evaluate mating disruption’s efficacy in controlling the OM from the first to the third generation. The effectiveness of MD formulations against the three generations of OM was assessed by determining the percentage of infested olive fruits, the reduction of pheromone trap catches, and the number of affected inflorescences in both MD-treated and untreated control olive groves. The number of release points (one or two aerosol devices per ha) was also evaluated. In all years and trials, the mean number of males caught in traps placed in the MD-treated plots was significantly lower than untreated sites. Mating disruption registered a high suppression of male captures (>75%) in treated plots for two consecutive seasons. Concerning infested olive fruits, substantial reductions (about 80%) were observed in the MD plots of locations B and C, and a reduction of about 40% was detected in location A, compared to the control plot. Results showed that the installation of two aerosol devices/ha reduced fruit damage below 20% of infested olive fruits except for one site where a reduction of about 71% in the MD plot was recorded in 2019. Although few significant differences were associated with OM male catches and infested olive fruits between plots treated with one aerosol/ha and two aerosols/ha in most of the comparisons, significant differences in the number of olive inflorescences infested by *P. oleae* were found, suggesting a similar performance between the two tested aerosol densities. Results of two-year field trials in Andalucía demonstrated the potential of Mister P X841 aerosol devices as an effective tool for controlling the olive moth, *P. oleae.*

## 1. Introduction

The olive moth (OM), *Prays oleae* (Bern.) (Lepidoptera: Yponomeutidae), is one of the most harmful and devastating olive grove pests in the Mediterranean Basin [1,2]. The damage caused by this moth can reduce production by 50–60%, causing heavy olive oil production losses [3,4]. *P. oleae* is a monophagous species that produces three generations per year [5,6,7]. Each generation is synchronized with the seasonal growth of specific plant structures—namely the leaves, flowers, and fruits [8].

During springtime, the first generation (phyllophagous) females lay eggs on the flower buds, from which larvae emerge (April–May), feed on the buds and flowers during full bloom, and initiate the second generation (anthophagous). The females of this generation lay eggs in the fruitlets, producing the third generation (carpophagous) which bore into the developing fruits (May–June), causing fruit drop and significant economic losses. After completing development, these larvae emerge from the fruits to pupate on branches, causing a second (September–October) fruit fall. The cycle is reinitiated when the females from the carpophagous generation lay eggs on the leaves [9].

*P. oleae* has been traditionally controlled using insecticides such as deltamethrin, lambda-cyhalothrin, acetamiprid, phosmet or—more recently—spinetoram [10]. However, the anthophagous and carpophagous generations are challenging to manage with insecticides, because—after hatching—the larvae live inside the buds, flowers, and fruits. In 1979, the *P. oleae* female sex pheromone, (Z)-7-tetradecenal was discovered by Campion et al. [11]. Since then, it has been extensively employed for monitoring purposes [12]. Mazomenos et al. [13], studied the potential use of (Z)-7-tetradecenal for the control of *P. oleae* through a mating disruption approach in olive crops, showing up to a 96% decrease in male catches in treated plots.

Mating disruption (MD) is widely used as an environmentally friendly pest control method in economically important crops. In contrast to the conventional methods, based on pesticides, MD is highly effective, specific, and does not leave toxic residues on the fruit [14]. Various release methodologies have been reported to induce MD, including passive dispensers, meso-dispensers, and aerosols [15,16]. Reservoir dispensers are passive release devices in which the pheromone is contained inside a polymeric matrix (polyethylene tubes, micro or nanoencapsulated formulations) which are commonly employed MD devices [16], placed in fields at 500 to 1000 per hectare (ha) [15]. In recent years, aerosol-based MD technologies have been developed. The aerosol dispenser consists of a pressurized metal device that holds the active ingredient solution and a programmable electronic device that releases the pheromone at specific times [17].

The aerosol dispensers [17,18] have been shown to reduce crop damage effectively. The use of these devices has grown exponentially, primarily because only 2–5 dispensers are needed per ha. Additionally, aerosol dispenser-based MD treatments have been successfully used to control *Cydia pomonella* (L.) [19,20,21,22], *Lobesia botrana* [23,24], *Grapholita molesta* [25], *Plodia interpunctella* [26], *Amyelois transitella* [27,28], and *Trichoplusia ni* [29].

Small plot trials evaluating the effectiveness of MD against *P. oleae* showed promising results in Greece [13] and Egypt [30]. In both studies, the MD formulations were released via passive reservoir dispensers. However, these studies utilized many dispensers, releasing substantial amounts of pheromone per ha. Thus, investigations that focus on reducing costs and optimizing pheromone usage to reduce the implementation costs associated with the MD technique are necessary.

The present study sought to explore the efficacy of MD against the three generations of *P. oleae* by deploying aerosol dispensers releasing (Z)-7-tetradecanal over two consecutive years in three olive groves in southern Spain. The technique’s effectiveness was assessed by counting the number of male OMs in the pheromone-baited traps and by comparing the inflorescence and fruit infestation rates in the MD-treated and untreated plots.

## 2. Materials and Methods

### 2.1. Field Locations

Field studies were conducted in three olive groves in the Andalucía region (Southern Spain) from March to October 2019 and 2020 (Table 1). The first site was located in the municipality of Morón de la Frontera (Sevilla province, location A), the second in Castro del Rio (Córdoba province, location B), and the third in Úbeda (Jaén province, location C). All plots contained Arbequina olive trees and were exposed to similar climatic conditions and managed under integrated production. The experiment was a complete block design with three experimental trials (Locations A, B, and C) and two MD treatments were evaluated within each site. Each experimental unit was divided into three homogeneously distributed plots in the internal part of the unit. Notably, the olive groves selected for the study reported moderate to high *P. oleae* infestation in the previous years.

### 2.2. Standard Crop Management

All olive groves have historically had medium to large infestations of *Prays oleae*. Therefore, spraying programs (Table 2) with *Bacillus thuringiensis* (Bt) and/or insecticides were being applied at location A and B; however, neither Bt nor insecticides were applied at location C during our study.

### 2.3. Aerosol Formulation and Distribution

The Mister P X841 (Biogard, CBC Iberia) aerosol dispenser with a programmable electronic device to release the active ingredient in the field over time was used in the present study. The aerosol dispenser consists of a pressurized aluminum can containing a solvent solution of (Z)-7-tetradecenal (<8% *w/w*) mixed with a propellant. The Mister P X841 aerosols were deployed at two-thirds the canopy height and spaced more than 100 m apart to achieve a density of 1 or 2 aerosols/ha. The aerosol devices were installed and programmed to release the pheromone following the manufacturer’s instructions. The Mister P X841 aerosols were applied before or during the beginning of the first *P. oleae* adult flight and evenly distributed in the treated plots.

### 2.4. Male Daily Flight Periodicity Assessment

Before starting the MD treatments, an automated trap camera monitored an olive grove in Iznájar (Córdoba, Andalucía, Southern Spain) with an abundant *P. oleae* population from October to November (Trapview, Hrusevje, Slovenia). As in Lucchi et al. [23], the camera was customized to take 48 photographs (one every 30 min) of a sticky plate inside a (Z)-7-tetradecenal baited trap every day. Males were identified in each picture by visual inspection, and the number caught was recorded. Based on that information, the Mister P X841s were programmed, following the manufacturer’s instructions, to release pheromone only during the pest’s sexually active period, when males were present.

### 2.5. Mating Disruption Experiment

In each trial and year, two plots under MD strategy (i.e., pheromone-treated) were compared to a plot (i.e., 0 aerosols/ha) where the grower employed a conventional strategy. The Mister P X841 devices were hung from the top of 2 m poles placed in the top third of the tree canopy. The devices were installed 100 m apart and deployed at two densities: 1 and 2 aerosols/ha. The control (0 aerosols/ha) was treated using the grower’s standard methods against *P. oleae* (Table 2).

During the first year of the experiments (2019) the aerosols were applied (on 13 March in location A and B and 26 March in location C) and during 2020 experiment, the aerosol devices were hung on 25 February, 30 March, and 31 March (Locations A, B, and C respectively). In both years, they were distributed evenly before or during the OM’s first flight in the experimental plots. The MD devices tested herein only require one application for the entire season.

### 2.6. Efficacy of the Treatments

The efficacy of the MD strategy on *P. oleae* control was evaluated using three parameters: number of moths caught by the pheromone-baited traps, damaged inflorescences with or without living forms, and percentage of infested fruits. The percentage of infested fruits was evaluated by the presence of viable eggs and/or living larvae inside the fruit. Dry or predated eggs were counted separately from the live ones.

#### 2.6.1. Attraction to Monitoring Traps

In all plots, the pest flights were monitored throughout the study period with pheromone baited traps. Trap catches in the MD area were compared with those recorded in the untreated area. The effects of MD treatment on the attraction of male OMs to the pheromone traps were examined by placing monitoring funnel traps (Oppennatur and Semiotrap SL, Jaén Spain) baited with *P. oleae* pheromone in polyethylene vial dispensers (Semiotrap SL, Jaén, Spain). Eighteen pheromone delta traps were installed at each location; six traps per plot were positioned more than 100 m apart, inside the MD-treated and control sites. Traps were hung inside the olive canopy 1.80 m above the ground, and lures were replaced every 45 days. Each trap had an insect-killing strip at the bottom. The traps were deployed before the pest’s first flight and checked weekly at all three locations.

The suppression ratio, expressed as a percentage, was calculated using the equation:

Suppression ratio = (Captures in control plot − Captures in MD plot)/(Captures in control plot) × 100.

#### 2.6.2. Inflorescence Damage Estimation

In 2019 and 2020, the OM anthophagous generation induced damage to inflorescences was estimated by visually identifying larvae and/or direct damage to the olive flower clusters. In each of the three plots (two MD and one control), samplings were performed by checking 210 inflorescences from 20 olive trees/plot (*n* = 3) around each pheromone trap. In the first assessment, a flower cluster was considered damaged when larvae or pupae were found, or the flowers were partially damaged.

#### 2.6.3. Olive Fruit Infestation

Fruit samplings were carried out to assess the OM infestations in the MD-treated and control plots. For the carpophagous generation, olive fruit infestation was estimated by visually inspecting for eggs followed by dissection under a binocular to determine the presence of larvae. For each plot, in 2019 and 2020, 20 randomly chosen olive trees around each pheromone trap were used for fruit sampling (May, carpophagous generation). Thirty-five fruits per tree (210 fruits per plot) were randomly collected around the canopy of each sampling plot. All fruits were dissected under a binocular to determine if OM larvae were present. The fruit was considered infested when live eggs or larval penetration were present. Data were expressed as infestation percentage.

### 2.7. Pheromone Release Rate

Pheromone release was estimated by the gravimetric method to determine the residual content of the pheromone in aerosol under field conditions. All the devices were weighed on a scale (Digital hanging scales AWS, 1 g precision, calibrated with an external weight for accuracy of measurements) three times each season. According to the composition label of the aerosols used, the pheromone is 8% *w/w* and was released daily according to the manufacturer’s programming. The amount of pheromone released was calculated after applying that percentage to the weight differences, assuming a perfect solution of the pheromone inside the formulation.

### 2.8. Data Analysis

The analysis of variance (ANOVA) followed by post-hoc Tukey’s or Student–Newman–Keuls test at *p* < 0.05 after a root square transformation were used to study the differences observed between MD and control plots of fit *p* < 0.05; Levene’s test, the goodness of fit (*p* < 0.05). Tukey’s HSD test (*p* < 0.05) was performed on the fruit and inflorescence infestation results to detect difference between treatments. All the statistical analyses were performed using SPSS v. 22 (IBM, Chicago, IL, USA).

## 3. Results

### 3.1. Daily Flight Periodicity Assessment of Male P. oleae

**Figure 1:**
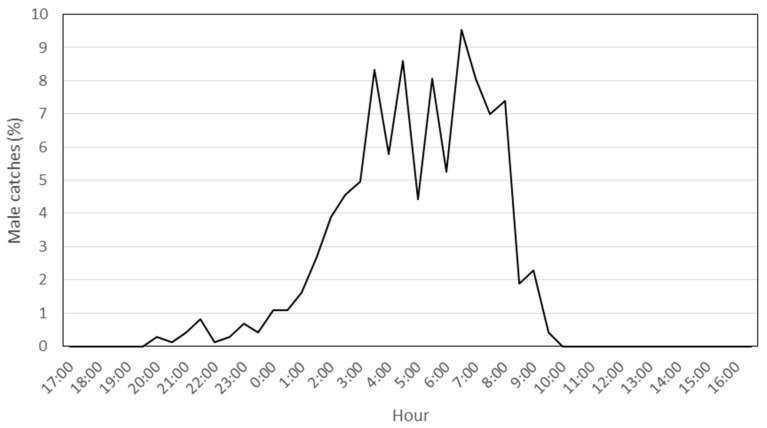
Daily male *P. oleae* device lure periodicity during the third generation. Percentages of male *P. oleae* catches/hour during the third generation in 2018.

After the preliminary monitoring period, during *P. oleae*’s third generation in 2018, 744 males were captured inside a single trap, a value within the normal range of captures in conventional funnel traps. As expected, most of the captures were between 1:00 and 9:00 a.m., a period when *P. oleae* are sexually active (Figure 1).

**Figure 2:**
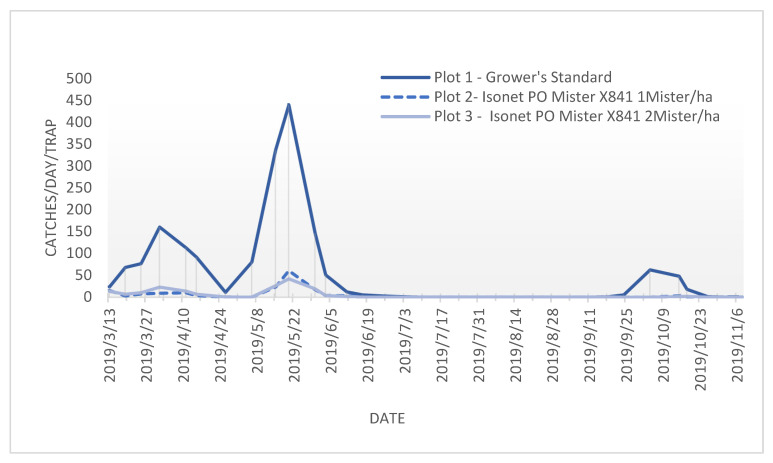
Flight periods of adult *Prays. oleae* with different treatments at location A from March to November 2019.

The annual flight period in all the trials and plots was similar, and it indicated that there were three succeeding *P. oleae* generations (Figure 2), with a small peak at the start of springtime (anthophagous generation), followed by a higher number of *P. oleae* adults flying halfway spring (carpophagous generation) and finally followed by a third flight period from the end of summer to the beginning of autumn (phyllophagous generation).

### 3.2. Attraction to Monitoring Traps and Suppression Ratio

Average catches per monitoring trap for the treated and control plots are displayed in Table 3. Compared to the control, significantly fewer male OMs were captured at the MD-treated trials throughout the season. In both years, and at all three plots, the total male catches using traps baited with the female sex pheromone component (i.e., (Z)-7-tetradecenal) showed significant differences among the three tested control strategies, with a higher abundance of male catches in the control plot compared to plots where one aerosol/ha and two aerosols/ha of Mister P X841 were deployed. In 2019, male catches varied significantly among the treatments: location A (F = 95.379; df = 2.15; *p* ≤ 0.001), location B (F = 46.820; df = 2.15; *p* ≤ 0.001) and location C (F = 30.052; df = 2.15; *p* ≤ 0.001). Similarly, in 2020, OM captures significantly differed between treatments, with a lower number of catches in the MD plots than in control plots, location A (F = 72.620; df = 2.15; *p* ≤ 0.001), location B (F = 207.620; df = 2.15; *p* ≤ 0.001), and location C (F = 36.802; df = 2.15; *p* ≤ 0.001).

There was a decreasing trend of OM captures as the number of aerosol applications increased. Moreover, in 2019—at locations B and C—significantly fewer moths were captured when two aerosols/ha were deployed than only one aerosol/ha. Regarding the 2020 season, differences in OM catches were observed between one aerosol/ha and two aerosols/ha at locations A and C but not location B.

The suppression ratio of male device lures was very high in all the trials (Table 4). For example, in location A the total catches of *P. oleae* during the complete second season of 2020 were reduced by 68.93% and 94.77% in plots treated with one and two aerosols/ha of pheromone compared to the control plot, respectively. It should be pointed out that even one aerosol/ha resulted in a relatively high suppression of catches. In 2019, the suppression ratio calculated for total capture ranged from 51.7% in location C, one aerosol/ha plot, to 91.71% in location A. Additionally, in 2020, the suppression ratio ranged from 68.93% to 85.55% in locations A and B, respectively. Notably, the capture suppression ratio exceeded 80% at the two aerosols/ha MD plots at all locations in both years. The relatively lower reduction (i.e., 63.93% in the plot with one aerosol/ha) observed during 2020 at location A was probably due to a mandatory change due to the loss of irrigation on the original plot.

Concerning trap captures by plot location (border or center) of the MD-treated olive grove, significant differences were observed in both years but not at all locations (Table 5). For example, in 2020 at location A, male catches varied significantly between moth catches at the center (F = 105.96; df = 2.6; *p* ≤ 0.001) and border (F = 130.08; df = 2.6; *p* ≤ 0.001).

### 3.3. Crop Damage Prospection: Inflorescences and Olive Fruit Infestation

Two parameters of *P. oleae* infestation levels were studied in the MD-treated and untreated plots: the percentage of damaged inflorescences for the anthophagous generation and the egg density and number of olive fruit penetrations for the carpophagous generation. As shown in Table 6, the results indicate an adequate general efficacy of the treatments with different significance levels depending on the year and plot.

The 2019 study results showed that the damage assessment for the anthophagous generation (i.e., the percentage of damaged inflorescences by *P. oleae*) was significantly reduced in locations A and B (F = 19.077; df = 2.15; *p* ≤ 0.001 and F = 14.528; df =2.15; *p* ≤ 0.001, respectively) on the MD-treated plots but not in location C (F = 0.004; df = 2.15; *p* = 0.996) where the OM moth population was low. In 2020, the inflorescence infestation was significantly lower in in all the treated locations (location A (F = 13.629; df = 2.15; *p* ≤ 0.001), location B (F = 95.460; df = 2.15; *p* ≤ 0.001) and location C (F = 94.475; df = 2.15; *p* ≤ 0.001)) compared to the untreated control plots.

Regarding the effect of MD on olive fruit infestation, in 2019 fruit damage was lower in all the MD plots (location A (F = 4.660; df = 2.15; *p* = 0.027), location B (F = 6.080; df = 2.15; *p* = 0.004), and location C (F = 1.922; df = 2.15; *p* ≤ 0.001)) than in the untreated control plots. In 2020, significantly lower fruit infestation was observed in the locations in B and C (F = 48.044; df = 2.15; *p* ≤ 0.001 and F = 18.164; df = 2.15; *p* ≤ 0.001, respectively) when comparing MD and control plots. In 2020, at location A, the effect of the treatments on fruit infestation was not statistically significant despite fruit infestation being reduced by almost half in the plot with two aerosols/ha.

More attenuated inflorescence and fruit damage were observed in MD plots treated with two aerosols/ha compared to one aerosol/ha (Table 6). However, these differences were not significant in most cases. On the other hand, significantly lower infestation levels were obtained compared to the control plot. In 3 out of 12 comparisons, the anthophagous generation at locations A and B and the carpophagous generation at location B, differences between the MD treatments were detected.

### 3.4. Pheromone Release Rate

**Figure 3:**
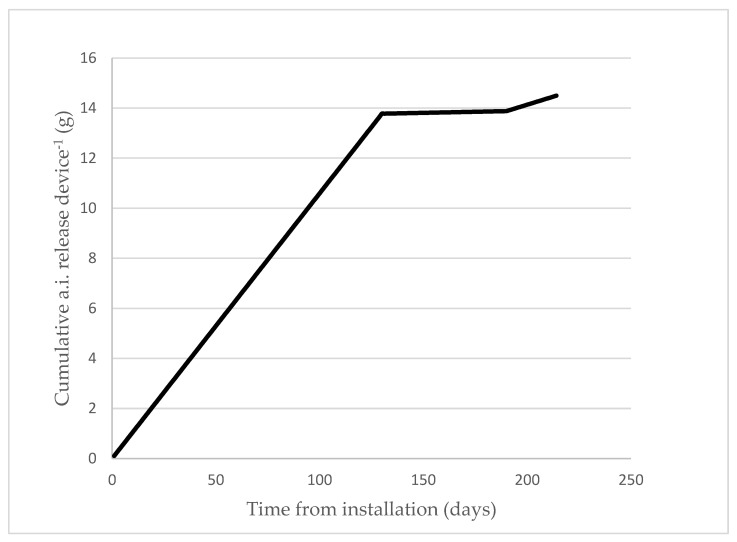
Estimated pheromone release (g/device) profile of the Mister P X841 aerosol devices at location A in 2020. Similar emission profiles were observed at the other locations in 2019 and 2020.

In order to determine the pheromone release rate of Mister P X841 MD dispensers, each year at three different dates during the trial, all the devices were weighed in the field and then re-hung again. The difference between the final weight and tare was around one-fourth the initial weight, indicating that approximately 75% of the pheromone loaded into the devices was released during the experimental period, assuming the release rate of pheromone and dispensers is consistent. The release curve of the sex pheromone demonstrated that the pheromone accumulates in the treated plots (Figure 3). It was estimated that the release of pheromone from the aerosol was approximately 90 mg/aerosol/day during the anthophagous and carpophagous generations, and 56 mg/aerosol/day for the phyllophagous generation, considering the residual pheromone from the aerosols.

## 4. Discussion

The effectiveness of MD in this study was assessed by counting the number of adults caught in the monitoring traps and examining inflorescences and fruit damage. A considerable reduction was observed in the number of male OMs caught in MD-treated plots compared to control plots in all trials. In addition, we showed that the percentage of damaged inflorescences and fruits was significantly higher in plots treated with the grower’s control pest treatment than in plots where Mister P X841s were deployed.

Our results showed a higher abundance of males caught in pheromone traps in untreated plots than in MD-treated plots in both years and all plots. Similar results using aerosol dispensers have been obtained for other pests such as *Cydia pomonella* [31,32] and *Lobesia botrana* [23]. However, the trap location (center or border) with the same treatment did not always significantly affect the male catches. At high population densities, males may move freely between plots, and can locate females using other cues [14].

Gravimetric methods that weigh dispensers at specified days over the season lack sufficient accuracy to establish pheromone release profiles for different dispensers [33], especially with aerosol devices. The two doses of synthetic sex pheromone used in this study, 16 and 32 g a.i./ha, resulted in high average (80.45% in 2019 to 83.80% in 2020) suppression ratios. As shown in Figure 3, at the end of the trial, the amount of pheromone released was 15 g a.i/ha using one P Mister X841/ha and 30 g a.i./ha when using two devices. However, the higher dose of pheromone (i.e., two Mister P X841/ha, 32 g a.i./ha) did not always significantly reduce male catches (Table 6). Similar results were obtained by McGhee et al. [31] for *C. pomonella,* in which no significant differences were found using higher doses.

Previous results reported by Hegazi et al. [34] showed that a mean rate of 20 mg/ha/day provided effective communication disruption. In an earlier field test on MD of *P. oleae* [13], a pheromone-cyclodextrin complex formulated in plastic bags was used, resulting in the inhibition of trap catches. Nevertheless, their results showed that MD was only effective in semi-isolated olive groves in terms of fruit infestation.

Another critical factor was the timing of pheromone application. In 2019 at location C, the first pheromone application was delayed one month after the first adults were caught in the monitoring trap because of operational problems. Moreover, the relatively low suppression ratio of males captured (51.76%) indicate the presence of a resident *P. oleae* population when this treatment started or probably because females had a chance to mate prior to the commencement of the MD treatments.

In 2019, Úbeda (plot C) was the only site where the percentage of inflorescences damaged in treated and control plots did not statistically differ for *P. oleae* infestation. During 2019 and 2020, significant differences in the fruit level—with variable efficacy depending on location—were found in the control and pheromone plots. Many factors—including population dynamics, field size, host species, dominant winds, and/or behavior of insect target species—can influence this pest control technique’s field efficacy [35,36]. It is important to point out that aerosol devices are often supplemented with a border application of hand-applied pheromone dispensers and/or companion insecticides, and in this trial, this was not the case.

Mating disruption using aerosol dispenser treatments in three Andalusian olive groves of the alberquina variety, consecutively for 2 years, clearly showed favorable effects on the population density of *P. oleae.* The results indicated that the deployment of Mister P X841 MD dispensers contributed to reducing olive fruit damage and the percentage of damaged inflorescences in all the trials. The high level of orientation disruption observed in all trials, irrespective of the number of olive trees/ha, showed that the efficacy of the aerosol MD treatments is not dependent on the planting pattern. Moreover, the life cycle of the OM may be influenced by several factors [1], causing wide variations in OM populations from year to year. On the other hand, the low number of devices per hectare (one or two) means we have developed an easy-to-apply technique that generates practically no waste in the environment. After testing, all aerosols are removed and each part is recycled, contributing to the zero-waste strategy.

Regarding the mode of production, this technique seems to work regardless of the type of crop management. It is generally known that larger treatment areas are desirable in MD [18], but our results demonstrate that a significant reduction of *P. oleae* infestation can be achieved in plots as small as 9–20 ha. Results of two-year field trials carried out in Andalucía demonstrated the potential of Mister P X841—as an effective tool for controlling the OM *P. oleae.*

## 5. Conclusions

In the present work, a MD system based on aerosol Mister P X841 MD dispensers was developed to control *P. oleae* populations. The treatment significantly reduced male catches comparted to the untreated olive crop plot throughout the complete *P. oleae* life cycle. Our results also demonstrated that MD using aerosol dispensers to manage *P. oleae* could reduce the inflorescence and the fruit infestation.

## Figures and Tables

**Table 1:** Features of the olive grove trials used to evaluate the efficacy of mating disruption against *Prays oleae* from 2019 to 2020.

Year	Olive Grove	Location	Trial Surface (ha)	Dose (Aerosols/ha)	Planting Pattern (x m Row Spacing and x m between Trees within Rows)
2019 and 2020	Location A	37°11′.01” N 5°29′22.35” W	Control plot: 12.70	Control plot: 0	(5 × 1.50)
			Plot 1: 17.50	Plot 1: 1	
			Plot 2: 14.80	Plot 2: 2	
	Location B	37°45′9.97” N 4°30′1.04” W	Control plot: 13.50	Control plot: 0	(3.75 × 1.35)
			Plot 1: 15.40	Plot 1: 1	
			Plot 2: 9.80	Plot 2: 2	
	Location C	37°54′4.01” N 3°13′58.74” W	Control plot: 29.70	Control plot: 0	(7 × 7)
			Plot 1: 20.30	Plot 1: 1	
			Plot 2: 9.08	Plot 2: 2	

**Table 2:** Conventional pest management methods used for the present study.

	Location A	Location B	Location C
2019	Spinetoram 25%	*Bacillus thurigiensis* (CEPA ABTS-351)	No treatment
2020	Spinetoram 25% + Acetamiprid 20%	No treatment	No treatment

**Table 3:** Number of *P. oleae* males caught per trap (mean ± SD) at the MD and control locations using different pheromone treatments, 2019–2020.

Year/Location	Generation	Control	1 Mister/ha	2 Mister/ha
2019				
Location A	First	3660.30 ± 1134.30 ^a^	277.80 ± 121.50 ^b^	464.70 ± 264.40 ^b^
	Second	7860.20 ± 1543.10 ^a^	738.20 ± 295.00 ^b^	673.00 ± 295.10 ^b^
	Third	1257.80 ± 490.60 ^a^	42.80 ± 27.30 ^b^	24.8 ± 38.10 ^b^
Total catches		12778.30 ± 2847.50 ^a^	1058.80 ± 389.10 ^b^	1162.50 ± 552.00 ^b^
F = 95.379; df = 2.15; *p* ≤ 0.001				
Location B	First	1397.30 ± 914.70 ^a^	349.20 ± 183.10 ^b^	57.80 ± 37.30 ^c^
	Second	4599.00 ± 879.90 ^a^	1095.20 ± 370.50 ^b^	507.50 ± 138.70 ^c^
	Third	696.80 ± 284.50 ^a^	121.70 ± 69.30 ^b^	57.70 ± 33.80 ^c^
Total catches		6693.20 ± 1978.30 ^a^	1566.10 ± 400.80 ^b^	623.00 ± 167.00 ^c^
F = 46.820; df = 2.15; *p* ≤ 0.001				
Location C	First	315.00 ± 57.70 ^a^	199.00 ± 127.20 ^b^	51.80 ± 8.00 ^c^
	Second	171.80 ± 14.20 ^a^	70.00 ± 20.90 ^b^	52.30 ± 22.60 ^b^
	Third	103.70 ± 162.50 ^a^	15.80 ± 14.00 ^b^	7.50 ± 4.20 ^b^
Total catches		525.66 ± 80.40 ^a^	284.80 ± 138.40 ^b^	111.70 ± 16.80 ^c^
F = 30.052; df = 2.15; *p* ≤ 0.001				
2020				
Location A	First	4956.00 ± 2117.50 ^a^	1458.00 ± 529.10 ^b^	303.00 ± 177.00 ^c^
	Second	6373.70 ± 1509.60 ^a^	2107.00 ± 690.90 ^b^	283.00 ± 194.30 ^c^
	Third	330.70 ± 79.30 ^a^	57.70 ± 13.50 ^b^	24.00 ± 11.70 ^c^
Total catches		10666.30 ± 2670.70 ^a^	3622.70 ± 981.30 ^b^	610.00 ± 229.00 ^c^
F = 72.089; df = 2.5; *p* ≤ 0.001				
Location B	First	5763.30 ± 2704.80 ^a^	1580.20 ± 378.20 ^b^	1576.30 ± 606.80 ^b^
	Second	11,480.30 ± 2581.00 ^a^	2077.20 ± 1112.70 ^b^	1335.00 ± 680.00 ^b^
	Third	74.30 ± 25.30 ^a^	10.20 ± 6.00 ^b^	3.20 ± 2.30 ^b^
Total catches		17318.00 ± 1662.00 ^a^	3667.5 ± 1261.80 ^b^	2914.50 ± 1159.20 ^b^
F = 207.620; df = 2.15; *p* ≤ 0.001				
Location C	First	496.80 ± 153.40 ^a^	171.30 ± 72.60 ^b^	128.20 ± 29.70 ^b^
	Second	1492.00 ± 451.70 ^a^	290.00 ± 170.70 ^b^	131.30 ± 41.80 ^c^
	Third	96.20 ± 48.70 ^a^	22.30 ± 26.40 ^b^	7.80 ± 6.40 ^c^
Total catches		2085.70 ± 643.60 ^a^	483.70 ± 255.20 ^b^	267.30 ± 53.80 ^c^
F = 36.802; df = 2.15; *p* ≤ 0.001				

Means followed by the same letter do not differ significantly (*p* < 0.05, Tukey test) between generations.

**Table 4:** Suppression ratio of *P. oleae* catches per location and MD dose in 2019 and 2020.

Year/Generation	Location A	Location B	Location C
2019			
One aerosol/ha	91.71%	76.60%	51.76%
Two aerosols/ha	90.90%	90.69%	81.09%
2020			
One aerosol/ha	68.93%	85.55%	76.80%
Two aerosols/ha	94.77%	83.14%	93.90%

**Table 5:** Total catches (mean ± SD) per trap per location within a plot (center or border).

2019					
	Location A				
	Border traps	Center traps	F	df	*p*
Control	10,767.00 ± 465.25 aA	14,789.66 ± 1579.52 aA	5.968	(1.4)	0.971
MISTERx1	757.00 ± 179.01 bB	1360.66 ± 54.69 bA	10.401	(1.4)	0.259
MISTERx2	1443.00 ± 406.26 bA	882.00 ± 101.10 bA	1.794	(1.4)	0.013
	F = 226.821	F = 74.556			
	df = 2.6	df = 2.6			
	*p* ≤ 0.001	*p* ≤ 0.001			
	Location B				
	Border traps	Center traps	F	df	*p*
Control	5534.00 ± 248.70 aA	7852.30 ± 1362.30 aA	2.803	(1.4)	0.169
MISTERx1	1818.00 ± 193.70 bA	1314.00 ± 181.20 bA	3.610	(1.4)	0.130
MISTERx2	761.7 ± 12.46 cA	484.30 ± 62.17 bB	19.130	(1.4)	0.012
	F = 226.821	F = 74.556			
	df = 2.6	df = 2.6			
	*p* ≤ 0.001	*p* ≤ 0.001			
	Location C				
	Border traps	Center traps	F	df	*p*
Control	592.30 ± 28.90 aA	459.00 ± 10.26 aB	18.924	(1.4)	0.012
MISTERx1	266.70 ± 59.81 bA	303.00 ± 109.80 abA	0.084	(1.4)	0.786
MISTERx2	117.60 ± 11.86 bA	105.70 ± 7.62 bA	0.724	(1.4)	0.443
	F = 38.828	F = 7.701			
	df = 2.6	df = 2.6			
	*p* ≤ 0.001	*p* = 0.022			
2020					
	Location A				
	Border traps	Center traps	F	df	*p*
Control	9520.60 ± 841.40 aB	13,800.0 ± 810.90 aA	13.411	(1.4)	0.022
MISTERx1	4004.00 ± 802.24 bA	3241.3 ± 116.20 bA	0.885	(1.4)	0.400
MISTERx2	552.7 ± 186.60 cA	667.3 ± 74.74 cA	0.325	(1.4)	0.599
	F = 44.279	F = 214.718			
	df = 2.6	df = 2.6			
	*p* ≤ 0.001	*p* ≤ 0.001			
	Location B				
	Border traps	Center traps	F	df	*p*
Control	17,289.00 ± 631.30 aA	17,347.00 ± 1379.30 aA	0.001	(1.4)	0.971
MISTERx1	4300.00 ± 953.20 bA	3035.00 ± 134.60 bA	10.401	(1.4)	0.259
MISTERx2	3872.00 ± 184.40 bA	1975.00 ± 411.07 bB	1.794	(1.4)	0.013
	F =130.076	F = 105.958			
	df = 2.6	df = 2.6			
	*p* ≤ 0.001	*p* ≤ 0.001			
	Location C				
	Border traps	Center traps	F	df	*p*
Control	2650.30 ± 143.10 aA	1519.70 ± 71.00 aB	50.071	(1.4)	0.002
MISTERx1	653.00 ± 159.50 bA	314.00 ± 11.40 bA	4.482	(1.4)	0.102
MISTERx2	270.30 ± 30.53 bA	264.30 ± 38.31 bA	0.015	(1.4)	0.908
	F = 104.53	F = 228.149			
	df = 2.6	df = 2.6			
	*p* ≤ 0.001	*p* ≤ 0.001			

Different lowercase letters indicate significant differences between treatments within the same trap location Tukey test (*p* < 0.05, Tukey test). Different capital letters indicate significant differences between trap locations within the same treatment (*p* < 0.05, Tukey test).

**Table 6:** Percentage (mean±SD) of inflorescences and olive fruits damaged by *P. oleae* larvae under different pheromone treatments in 2019 and 2020.

Year	Location	Treatment (Aerosols/ha)	% Damaged Inflorescences	% Infested Fruits
2019	A	Control	52.50 ± 8.14 a	47.97 ± 5.21 a
		PO-MISTERx1	14.17 ± 2.71 b	26.76 ± 4.58 b
		PO-MISTERx2	10.83 ± 3.27 b	25.75 ± 7.28 b
			F = 19.077	F = 4.660
			df = 2.15	df = 2.15
			*p* ≤ 0.001	*p* = 0.027
	B	Control	25.83 ± 6.11 a	14.14 ± 1.01 a
		PO-MISTERx1	4.17 ± 1.53 b	6.06 ± 2.59 b
		PO-MISTERx2	0.00 ± 0.00 b	4.54 ± 1.51 b
			F = 14.528	F = 6.080
			df = 2.15	df = 2.15
			*p* ≤ 0.001	*p* = 0.004
	C	Control	35.83 ± 4.90 a	32.83 ± 5.97 a
		PO-MISTERx1	36.67 ± 1.66 a	8.58 ± 3.44 b
		PO-MISTERx2	36.67 ± 12.42 a	5.56 ± 2.13 b
			F = 0.004	F = 1.922
			df = 2.15	df = 2.15
			*p* = 0.996	*p* ≤ 0.001
2020	A	Control	27.14 ± 3.36 a	41.45 ± 8.00 a
		PO-MISTERx1	15.71 ± 2.83 b	34.75 ± 9.57 a
		PO-MISTERx2	5.71 ± 2.44 c	24.93 ± 3.88 a
			F = 13.629	F = 1.922
			df = 2.15	df = 2.15
			*p* ≤ 0.001	*p* = 0.181
	B	Control	58.10 ± 2.51 a	44.76 ± 2.52 a
		PO-MISTERx1	26.19 ± 0.87 b	24.76 ± 3.27 b
		PO-MISTERx2	24.29 ± 2.05 b	9.04 ± 1.71 c
			F = 95.460	F = 48.044
			df = 2.15	df = 2.15
			*p* ≤ 0.001	*p* ≤ 0.001
	C	Control	45.24 ± 1.36 a	33.33 ± 3.35 a
		PO-MISTERx1	21.90 ± 1.75 b	14.76 ± 1.71 b
		PO-MISTERx2	16.67 ± 1.55 c	18.09 ± 1.41 b
			F =94.475	F = 18.164
			df = 2.15	df = 2.15
			*p* ≤ 0.001	*p* ≤ 0.001

Means followed by the same lowercase letter in each row are not significantly different (*p* < 0.05, Tukey test).

## Data Availability

The data presented in this study are available on request from the corresponding author.
